# A comparative analysis of the vibrational behavior of various beam models with different foundation designs

**DOI:** 10.1016/j.heliyon.2024.e26491

**Published:** 2024-02-23

**Authors:** Gulnaz Kanwal, Naveed Ahmed, Rab Nawaz

**Affiliations:** a Department of Mathematics, COMSATS University Islamabad, Park Road, Tarlai Kalan, 45550, Islamabad, Pakistan; b Department of Mathematics and Natural Sciences, Centre for Applied Mathematics and Bioinformatics, Gulf University for Science and Technology, 32093 Hawally, Kuwait

**Keywords:** Timoshenko beam, Pasternak foundation, Hetényi foundation, Vibration frequency, Galerkin finite element method

## Abstract

This article discusses the modal behavior of elastically constrained beams under various types of foundations and provides insights into the effects of different factors on the eigenfrequencies of beams. Numerical and analytical techniques, specifically the Galerkin finite element method (GFM) and the separation of variables, are utilized to determine the eigenfrequencies and mode shapes of beams. Modal analysis of Timoshenko, shear, Rayleigh, and Euler-Bernoulli beams that are elastically constrained and resting on Winkler, Pasternak, and Hetényi foundations, considering non-classical boundary conditions, is included in the study. The effects of factors such as flexural rigidity, transverse modulus, and Winkler foundation constant on natural frequencies of different beam models are investigated. The proposed method efficiently converges to the exact solution without shear locking in the stiffness element. The results demonstrate that the natural frequencies of the beam rise because of the shear layer, flexural rigidity, and foundation constant. Furthermore, the Hetényi elastic foundation affects the natural frequency of the beam, depending on the relative values of beam stiffness and foundation stiffness. Additionally, incorporating both shear deformation and rotary inertia has a greater impact on the eigenfrequencies of Euler-Bernoulli beams compared to incorporating only one of these effects. The findings of this work provide valuable insights into the behavior of beams under different foundation conditions and have potential applications in the design and optimization of structures incorporating beams, thereby enhancing the understanding of beam analysis.

## 1 Introduction

Beams are widely used in machines and structures for their ability to transmit loads and resist bending, shear, and torsion forces. They provide structural support in buildings, bridges, and towers, and they are used in various machine components such as frames, shafts, and linkages. Beams also play a vital role in transportation vehicles like cars, airplanes, and ships by distributing loads from the vehicle body to the wheels, wings, or hull. Material handling systems like cranes and conveyor belts utilize beams to support heavy loads and transfer them from one location to another. Additionally, beams are used in medical equipment like X-ray machines and radiation therapy devices, where they focus beams of radiation onto specific areas of the body while minimizing exposure to healthy tissue. Therefore, understanding the behavior of beams and selecting the appropriate materials and design is crucial in ensuring the reliability and safety of the machines and structures that use them.

Different beam theories are used to describe the behavior of beams under various conditions. Euler-Bernoulli beam (EBB) theory is used for long and thin beams, while Timoshenko beam (TB) theory is used for short and thick beams while shear beam(SB) theory is used for composite materials and Rayleigh beam (RB) takes into account rotatory inertia effect in addition to bending deformation, making it more accurate than the classical EBB theory in describing the behavior of such beams. These theories play an important role in the design and analysis of various structures and machine elements. The appropriate theory to use depends on the specific application and the type of loading that the beam is subjected to. Understanding the assumptions and limitations of each theory is essential in confirming the accuracy and reliability of the results. Also, beam vibrations are investigated considering beam dynamical phenomenon when a beam is placed on a surface that can deform under load, i.e.; elastic foundation. The elastic foundation can be soil, a concrete slab, or any other material that has some degree of flexibility. When the beam is subjected to external forces or self-excited forces, it can start to vibrate, and the vibration can be transmitted to the elastic foundation. Beam vibrations on elastic foundations are considered to be one of the most significant topics in structural engineering. Concrete structures and civil engineering structures are examples of structures on elastic foundations used as parts of machinery for isolation. Winkler, Pasternak, Vlasov, and Flonenko-Borodich foundations are a few examples of structures that are supported along their main axes.

Vibrating beam models without elastic foundations have been extensively investigated. An elastic beam with one end elastically constrained and the other end free was analyzed by Chun [1]. Lee [2] determined the characteristic equation for a beam with a rotating spring and a mass attached at one end. An analysis of beam dynamics is performed by Lai et al. [3] using the Adomian decomposition method. The sinc-Galerkin method was developed by Smith et al. [4] for solving beam problems with fixed boundary conditions. Sinc discretization appears to be the most effective method for obtaining numerical results. The vibrating beam's symmetrical spring-hinged ends caused Hess [5] to extend the inquisition. A Timoshenko beam's vibration analysis under non-classical boundary conditions has been researched by Abbas [6]. The Fourier technique was adopted by Kim and Kim [7] to ascertain the eigenvalues of the elastically constrained beam. The dynamic behavior of the damped beam with elastic constraints was examined by Mahapatra and Panigrahi [8] using the Fourier cosine series. A forced and free vibration of a double beam with arbitrary end conditions connected to a viscoelastic layer and discrete points is investigated by Zhao and Chang [9]. By using stress-based FEM, Wieckowski and Światkiewicz [10] proposed to solve the static bending problem for homogeneous Euler-Bernoulli and Timoshenko beams.

In [11], [12], [13], [23], [27], [28], [29] and the references herein, the interaction of structures with various foundations has been addressed in precise detail. Hsu [13], Shin et al. [14] and Rosa [15] used an elastic Winkler foundation (WF) that contains a single parameter. Hetenyi [16] dealt with the problems of uniform Euler-Bernoulli beams supported by an elastic Winkler foundation. Doyle and Pavlovic [17] performed a Vibrational analysis of beams resting on elastic partial foundations has been performed by Doyle and Pavlovic [17]. Rao investigated a clamped-clamped (C-C) homogeneous beam on intermediate elastic support [18]. The differential transform technique was employed by Kacar et al. [19] to investigate the vibration response of a beam placed over an elastic variable Winkler foundation.

The Pasternak foundation (PF) and Hetényi foundation (HF), which have transverse modulus or shear and flexural rigidity, respectively, are well known as a two-parameter foundation model. The initial parameter of the foundation in two-parameter foundation models is still the elastic Winkler foundation parameter. Shin et al. [14], Arboleda-Monsalve et al. [20], Zhu and Leung [21], and Civalek [22] described the Pasternak elastic foundation. Wang and Stephens [23] determined the eigenfrequencies of a Timoshenko beam supported by the Pasternak foundation (PF), and they also obtained the characteristic equations for various boundary conditions. El-Mously [24] utilized Rayleigh's concept to determine the Timoshenko beams' natural frequencies over the elastic Pasternak support. Lee et al. [25] explored the dynamic analysis of a beam over an elastic Pasternak foundation. The Timoshenko beam solution on a variable elastic basis was examined by Ghannadiasl and Mofid by using Green's functions [26].

The underlying study outlines the use of the Galerkin finite element method (GFM) and the separation of variables method for analyzing various types of beams (including Timoshenko, shear, Rayleigh, and Euler-Bernoulli beams) that are elastically constrained and resting on different types of foundations (Winkler, Pasternak, and Hetényi). The study includes modal analysis of these beams with both single and two parametric foundations and examines the influence of factors such as flexural rigidity, transverse modulus, and the Winkler foundation constant on the natural frequencies of the different models. Non-classical boundary conditions are also taken into account in the proposed models, as opposed to the commonly studied classical boundary conditions. It is worth mentioning that the underlying research is innovative in its utilization of non-classical boundary conditions and seeking more efficient and accurate solutions to the underlying problem. The GFM is shown to converge efficiently to the exact solution without any shear locking in the derived stiffness element. The research further reveals that the presence of a shear layer, flexural rigidity, and foundation constants leads to an increase in natural frequencies. While recent research has delved into numerical solutions addressing the influence of shear and rotary effects on structures resting on Winkler or Pasternak foundations [27], [29], this study distinguishes itself by simultaneously considering four engineering theories of beams, each with non-classical constraints, and the effects of various foundations. This comprehensive approach sets it apart from previous works. The primary objective is to establish an effective interplay between multiple factors. Notably, the choice of foundation type whether Winkler, Pasternak, or Hetényi exerts a substantial impact on the natural frequency of the beam, with the relative stiffness of both the beam and the foundation emerging as a pivotal determinant in this context. This study's distinct focus on these aspects enhances our understanding of structural behavior under varying conditions, contributing valuable understandings to the field. Additionally, incorporating both shear deformation and rotary inertia affects the eigenfrequencies of Euler-Bernoulli beams more than considering only one of these effects. As a result, the study offers valuable insights into beam behavior under diverse foundation conditions, opening avenues for future research in higher beam theories, modified models, and exploring the effects of various foundations and boundary conditions. These findings enhance our understanding of beam analysis and its applications in structural design and optimization.

The article is structured as follows. Section 2 incorporates a statement of the problem. Section 3 states a working procedure for calculating, eigenfrequencies, eigenvalues, and eigenmodes. Results and discussions are provided in Section 4, whereas Section 5 contains the conclusion.

## 2 Problem formulation

**Figure 1:**
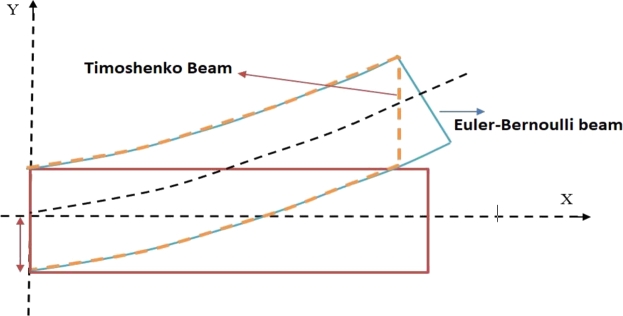
Timoshenko and Euler-Bernoulli beam configuration.

**Figure 2:**
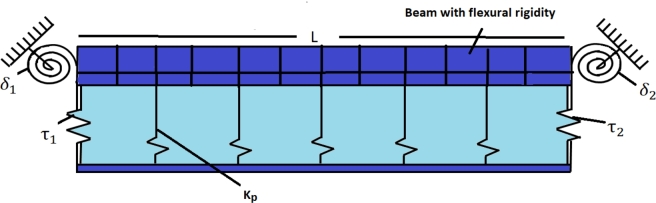
Timoshenko model resting over Hetényi foundation.

**Figure 3:**
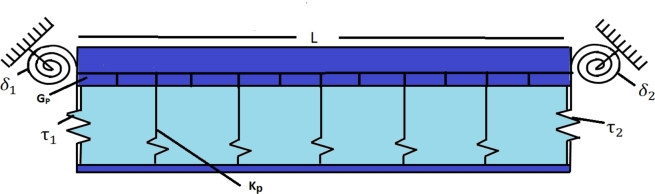
Timoshenko model resting over Pasternak foundation.

The underlying problem involves analyzing the transverse vibration of a TB as shown in Fig. 1, which considers both the effects of rotary inertia and shear deformation. Unlike the Euler-Bernoulli beam model, the planes in a Timoshenko beam are not perpendicular to the neutral axis of the beam. The beam is subjected to elastic constraints and is placed on the Hetényi and Pasternak elastic foundations, as shown in Figure 2, Figure 3, respectively. The problem involves solving the following set of coupled differential equations to determine the beam's natural frequencies and modes of vibration for various two-parametric elastic foundations.

(2.1a) $$\rho A\left(\frac{\partial^{2}\nu }{\partial t^{2}}\right)-KGA\left(\frac{\partial^{2}\nu }{\partial x^{2}}-\frac{\partial \psi }{\partial x}\right)+K_{p}\nu (x,t)-q\left(\frac{\partial^{2}\nu }{\partial x^{2}}\right)=0$$

(2.1b) $$-EI\frac{\partial^{2}\psi }{\partial x^{2}}-KGA\left(\frac{\partial \nu }{\partial x}-\psi \right)+\rho I\left(\frac{\partial^{2}\psi }{\partial t^{2}}\right)=0$$

where *ρ*, *A*, *I*, ν(x,t), and *E* are the mass density, cross-section area, second moment of inertia, displacement, and Young's modulus of the beam, respectively. K, G, *ψ*, and $K_{p}$ are the cross-section shape factor, modulus of rigidity, bending slope, and Winkler foundation constant, respectively. Generally, for a two-dimensional Pasternak elastic foundation, $q=G_{P}$, while q=−EI for Hetényi elastic foundation. Where $G_{P}$ is the shear foundation modulus (SFM), and EI is the flexural rigidity. However, if q=0 is taken into consideration, the governing equation for a Timoshenko beam resting on a Winkler foundation is obtained.

After eliminating *ν* and *ψ* from Eqs. (2.1a)–(2.1b), Eqs. (2.2a)–(2.2b) are obtained in terms of *ν* and *ψ*, respectively.

(2.2a) $$EI\frac{\partial^{4}\nu }{\partial x^{4}}+\rho A\frac{\partial^{2}\nu }{\partial t^{2}}-\frac{EI\rho }{KG}\frac{\partial^{4}\nu }{\partial x^{2}\partial t^{2}}-\rho I\frac{\partial^{4}\nu }{\partial x^{2}\partial t^{2}}+K_{p}\nu (x,t)-q\frac{\partial^{2}\nu }{\partial x^{2}}=0$$

(2.2b) $$EI\frac{\partial^{4}\psi }{\partial x^{4}}+\rho A\frac{\partial^{2}\psi }{\partial t^{2}}-\frac{EI\rho }{KG}\frac{\partial^{4}\psi }{\partial x^{2}\partial t^{2}}-\rho I\frac{\partial^{4}\psi }{\partial x^{2}\partial t^{2}}+K_{p}\psi (x,t)-q\frac{\partial^{2}\psi }{\partial x^{2}}=0$$

The homogeneous partial differential equations for *ψ* and *ν* are the same since they can only be decoupled when cross-section area and density are uniform. There are the following relevant boundary conditions for the elastically constrained TB:

(2.3a) $$-KGA\left(\frac{\partial \nu (0,t)}{\partial x}-\psi \right)=-\tau_{1}\nu (0,t)$$

(2.3b) $$-KGA\left(\frac{\partial \nu (L,t)}{\partial x}-\psi \right)=\tau_{2}\nu (L,t)$$

(2.3c) $$EI\frac{\partial \psi (0,t)}{\partial x}=\delta_{1}\psi (0,t)$$

(2.3d) $$EI\frac{\partial \psi (L,t)}{\partial x}=-\delta_{2}\psi (L,t)$$

where $\tau_{1}$, $\tau_{2}$, $\delta_{1}$, and $\delta_{2}$ represent translation and rotational spring constants at the left and right ends, respectively, of Timoshenko beams. Intriguingly, the shear beam, Rayleigh beam, and Euler-Bernoulli beams can be obtained as special cases once the rotary inertia and the shear deformation effects are eliminated from Eqs. (2.1a)-(2.1b).

**Case 1:** Equations (2.1a)-(2.1b) refer to a shear beam supported by single and two-parametric foundations.

**Case 2:** After eliminating the shear deformation effect, Eqs. (2.1a)-(2.1b) results in the Rayleigh beam resting over Winkler, Pasternak, and Hetényi elastic foundations.

**Case 3:** Once rotary inertia and shear deformation effects have been eliminated, Eqs. (2.1a)-(2.1b) yield the Euler-Bernoulli beam with various elastic foundations.

Additionally, the boundary conditions (2.3a)-(2.3d) deduce classical boundary conditions by varying spring constants. The following sections describe the analytical and numerical procedures for determining eigenmodes and eigenfrequencies.

## 3 Determination of natural frequencies and eigenmodes

This section provides a description of the procedure for determining the eigenfrequencies and eigenmodes. For approximate solutions to such problems, many researchers have employed a variety of techniques with some limitations and compromises. In order to find frequency relations and eigenfunctions analytically, we suggest separating the variables. Root-finding methods are employed to ascertain the eigenvalues and eigenfunction of the corresponding eigenmodes. A numerical solution and its validation are also established using the finite element scheme. Thus, we present solutions for the Timoshenko beam placed over various foundations in the following sections by illustrating analytical and numerical techniques.

### 3.1 Analytical solutions

To solve the coupled differential Eqs. (2.1a)-(2.1b), the approach of separating the variables is utilized. This involves assuming that the time solution, T(t), is separable from the spatial solutions for the bending slope and beam displacement. In other words, the bending slope and beam displacement are considered to be functions of position only. Furthermore, the time-synchronization of the bending slope and beam displacement ensures that they vary simultaneously, resulting in a physically meaningful solution.

(3.1) $$\left[\begin{array}{c} \nu \left(x,t\right)\\ \psi \left(x,t\right) \end{array}\right]=T(t)\left[\begin{array}{c} X\left(x\right)\\ \zeta \left(x\right) \end{array}\right]$$

Now, substituting the Eq. (3.1) into Eqs. (2.1a)–(2.1b) lead to

(3.2a) $$\rho AXT^{\prime \prime }-(KGAX^{\prime \prime }-KGA\zeta^{\prime }-K_{p}X+qX^{\prime \prime })T=0$$

(3.2b) $$EI\zeta^{\prime \prime }T+KGA(X^{\prime }-\zeta )T+\rho IXT^{\prime \prime }=0$$

The aforementioned equations are divided by the *XT* and *ζT*, respectively. The expressions for Eqs. (3.2a) and (3.2b) can be rewritten as:

(3.3a) $$\frac{T^{\prime \prime }}{T}=\frac{KGX^{\prime \prime }}{\rho X}+\frac{KG\zeta^{\prime }}{\rho X}+\frac{K_{p}}{\rho A}-\frac{qX^{\prime \prime }}{\rho AX}$$

(3.3b) $$\frac{T^{\prime \prime }}{T}=-\frac{E\zeta^{\prime \prime }}{\rho \zeta }-\frac{KGAX^{\prime }}{\rho I\zeta }+\frac{KGA}{\rho I}$$

Due to the separation of the variables, each side of Eqs. (3.3a) and (3.3b) must equal a constant, say $-\omega^{2}$ (natural frequency). As a result, we could write:

(3.4) $$T^{\prime \prime }+\omega^{2}T=0\;X^{\prime \prime }(KGA+q)-KGA\zeta^{\prime }-K_{p}X+\omega^{2}\rho AX=0$$

(3.5) $$T^{\prime \prime }+\omega^{2}T=0\;EI\zeta^{\prime \prime }+KGA(X^{\prime }-\zeta )+\omega^{2}\rho I\zeta =0$$

In Eqs. (3.2a)–(3.5), the second spatial equation can be expressed in the form of the following matrix notation.

(3.6) $$\left[\begin{array}{cc} KGA+q\; & 0\\ 0\; & EI \end{array}\right]\left[\begin{array}{c} X^{\prime \prime }\left(x\right)\\ \zeta^{\prime \prime }\left(x\right) \end{array}\right]+\left[\begin{array}{cc} 0\; & -KGA\\ KGA\; & 0 \end{array}\right]\left[\begin{array}{c} X^{\prime }\left(x\right)\\ \zeta^{\prime }\left(x\right) \end{array}\right]+\left[\begin{array}{cc} \omega^{2}\rho A-K_{p}\; & 0\\ 0\; & -KGA+\omega^{2}\rho I \end{array}\right]\left[\begin{array}{c} X\left(x\right)\\ \zeta \left(x\right) \end{array}\right]=0$$

decoupling of these equations will lead to

(3.7) $$EIX^{iv}+\frac{EIqX^{iv}}{KGA}-G_{P}X^{\prime \prime }+K_{p}X-\frac{K_{p}EIX^{\prime \prime }}{KGA}-\omega^{2}\rho AX+\frac{\omega^{2}\rho AEIX^{\prime \prime }}{KGA}+\frac{\omega^{2}\rho IqX^{\prime \prime }}{KGA}+\frac{\omega^{2}K_{p}\rho IX}{KGA}-\frac{\omega^{4}\rho^{2}AIX}{KGA}+\frac{\omega^{2}\rho IX}{KGA}=0\;EI\zeta^{iv}+\frac{EIq\zeta^{iv}}{KGA}-G_{P}\zeta^{\prime \prime }+K_{p}\zeta -\frac{K_{p}EI\zeta^{\prime \prime }}{KGA}-\omega^{2}\rho A\zeta +\frac{\omega^{2}\rho AEI\zeta^{\prime \prime }}{KGA}+\frac{\omega^{2}\rho Iq\zeta^{\prime \prime }}{KGA}+\frac{\omega^{2}K_{p}\rho I\zeta }{KGA}-\frac{\omega^{4}\rho^{2}AI\zeta }{KGA}+\frac{\omega^{2}\rho I\zeta }{KGA}=0$$

It is observed that the fourth-order homogeneous differential equations for *X* and *ζ* have similar forms, indicating that the solution to Eq. (3.7) can be acquired as a constant multiple.

(3.8) $$\left[\begin{array}{c} X\left(x\right)\\ \zeta \left(x\right) \end{array}\right]=d\text{V}e^{rx}$$

where **V**, *d*, and *r* are eigenvector, constant number, and eigenvalues. Now, Eq. (3.8) can be utilized in Eq. (3.6), to get

(3.9) $$\left[\begin{array}{cc} KGAr^{2}+\rho \;A\omega^{2}+qr^{2}-K_{p}\; & -KGAr\\ KGAr\; & IEr^{2}-KGA+\omega^{2}\rho I \end{array}\right]\text{V}=0$$

By enforcing a matrix determinant identical to zero, eigenvectors (V) and eigenvalues (r) are found using Eq. (3.9). Thus, we obtain

(3.10) $$r^{4}+\frac{r^{2}}{{\left(KGAEI+qEI\right)}^{2}}\left(-KGAq+\rho A\omega^{2}EI-K_{p}EIKGA+KGA\omega^{2}\rho I+q\rho I\omega^{2}\right)+\frac{1}{KGAEI+qEI}\left(-KGA^{2}\rho \omega^{2}+\omega^{4}\rho^{2}AI+K_{p}KGA-K_{p}\omega^{2}\rho I\right)=0$$

As a consequence, the eigenvalues can be determined from Eq. (3.11):

(3.11) $$r_{i}^{2}=\pm (E_{1}+E_{2})+\sqrt{{\left(E_{1}-E_{2}\right)}^{2}+E_{3}}\;\;\;i=1,\;2,\;3,\;4$$

where

(3.12) $$E_{1}=\frac{KGA\omega^{2}\rho I-KGAq+q\omega \rho I-K_{p}EI}{2(KGAEI+qEI)},\;\;E_{2}=\frac{EIA\rho \omega^{2}}{KGAEI+qEI},E_{3}=\frac{1}{{\left(KGAEI+qEI\right)}^{2}}(K^{2}G^{2}A^{3}\rho EI\omega^{2}-K_{p}K^{2}G^{2}A^{2}EI-K_{p}qKGAEI+K_{p}\omega^{2}\rho I^{2}EIKGA-K_{p}qEI^{2}\omega^{2}\rho -K_{p}E^{2}I^{2}\omega^{2}\rho A)$$

and corresponding eigenvectors $V_{i}$ are given in Eq. (3.13):

(3.13) $$V_{i}=\left[\begin{array}{c} KGAr_{i}\\ KGA{r_{i}}^{2}+\rho \;A\omega^{2}+q{r_{i}}^{2}-K_{p} \end{array}\right]\;\;\;or\;\;\;\left[\begin{array}{c} EIr_{i}^{2}-KGA+\rho I\omega^{2}\\ -KGAr_{i} \end{array}\right]$$

Spatial solution to the Eq. (3.10) is written as

(3.14) $$\left[\begin{array}{c} X\left(x\right)\\ \zeta \left(x\right) \end{array}\right]=\sum_{i=1}^{4}d_{i}V_{i}e^{r_{i}x}=d_{1}V_{1}e^{\beta x}+d_{2}V_{2}e^{-\beta x}+d_{3}V_{3}e^{i\alpha x}+d_{4}V_{4}e^{-i\alpha x}$$

where

$$\alpha^{2}=(E_{1}+E_{2})+\sqrt{{\left(E_{1}-E_{1}\right)}^{2}+E_{3}},\;\;\beta^{2}=-(E_{1}+E_{2})+\sqrt{{\left(E_{1}-E_{1}\right)}^{2}+E_{3}}$$

Eq. (3.14) can be reformulated as follows using sinusoidal and hyperbolic functions:

(3.15) $$\left[\begin{array}{c} X\\ \zeta \end{array}\right]=\left[\begin{array}{c} A_{1}\\ B_{1} \end{array}\right]sin(\alpha x)+\left[\begin{array}{c} A_{2}\\ B_{2} \end{array}\right]cos(\alpha x)+\left[\begin{array}{c} A_{3}\\ B_{3} \end{array}\right]sinh(\beta x)+\left[\begin{array}{c} A_{4}\\ B_{4} \end{array}\right]cosh(\beta x)$$

Here, $A_{1}$, $A_{2}$, $A_{3}$, $A_{4}$, $B_{1}$, $B_{2}$, $B_{3}$, and $B_{4}$, are constants. The eight constant in Eq. (3.15) appear to be unknown, so we can relate $A_{i}$ and $B_{i}$ from Eq. (3.16):

(3.16) $$B_{1}=\frac{-\alpha^{2}(KGA+q)+(-K_{p}+\rho A\omega^{2})}{KGA\alpha }A_{2}\;B_{2}=\frac{-\alpha^{2}(KGA+q)+(-K_{p}+\rho A\omega^{2})}{-KGA\alpha }A_{1}\;B_{3}=\frac{\beta^{2}(KGA+q)+(-K_{p}+\rho A\omega^{2})}{KGA\beta }A_{4}\;B_{4}=\frac{\beta^{2}(KGA+q)+(-K_{p}+\rho A\omega^{2})}{KGA\beta }A_{3}$$

Now, Eq. (3.15) is left with four unknowns. These relations can be obtained more easily by substituting the assumed solution (3.15) into the spatial differential equations (3.6). Using Eq. (3.15) into Eq. (2.3a)-(2.3d), we obtain

(3.17) $$A_{1}(\alpha -(\frac{-\frac{\gamma^{2}\alpha^{2}q}{AGK}-\frac{\gamma^{2}K_{p}}{AGK}-\frac{\gamma^{2}q}{EI}+\gamma^{2}\beta^{2}Z+\alpha^{2}Z}{\alpha (\gamma^{2}+Z)}+\frac{\alpha q}{AGK}+\frac{K_{p}}{\alpha (AGK)}))-A_{2}(\frac{\tau_{1}}{AGK})+A_{3}(\beta -(\frac{-\frac{\gamma^{2}\beta^{2}q}{AGK}+\frac{\gamma^{2}K_{p}}{AGK}+\frac{\gamma^{2}q}{EI}+\gamma^{2}\alpha^{2}+\beta^{2}Z}{q(\gamma^{2}+Z)}+\frac{\beta q}{AGK}-\frac{K_{p}}{\beta (AG\;K)}))-A_{4}\frac{\tau_{1}}{AGK}=0$$

(3.18) $$A_{1}(-\frac{\delta_{1}}{EI}(\frac{-\frac{\gamma^{2}\alpha^{2}q}{AGK}-\;\frac{\gamma^{2}K_{p}}{AGK}-\frac{\gamma^{2}q}{+}\gamma^{2}\beta^{2}Z+\alpha^{2}Z}{\left(\alpha (\gamma^{2}+Z\right)}+\frac{\alpha q}{AGK}+\frac{K_{p}}{\alpha (AGK)}))EI)+A_{2}(\frac{\frac{\gamma^{2}\alpha^{2}q}{AGK}+\frac{\gamma^{2}K_{p}}{AGK}EI+\frac{\gamma^{2}\;q}{EI}-\gamma^{2}\beta^{2}Z-\alpha^{2}Z}{\gamma^{2}+Z}-\frac{\alpha^{2}q}{AGK}-\frac{K_{p}}{AGK})+A_{3}(-\frac{\delta_{1}}{EI}(\frac{-\frac{\gamma^{2}\beta^{2}q}{AGK}+\frac{\gamma^{2}K_{p}}{AGK}+\frac{\gamma^{2}q}{EI}+\gamma^{2}\alpha^{2}+\beta^{2}Z}{\beta (\gamma^{2}+Z)}+\frac{\beta q}{AGK}-\frac{K_{p}}{\beta (AGK)}))-A_{4}(\frac{-\frac{\gamma^{2}\beta^{2}q}{AGK}+\frac{\gamma^{2}K_{p}}{AGK}+\frac{\gamma^{2}q}{EI}+\gamma^{2}\alpha^{2}Z+\beta^{2}Z}{\gamma^{2}+Z}+\frac{\beta^{2}q}{AGK}-\frac{K_{p}}{AGK})=0$$

(3.19) $$A_{1}(\cos(\alpha L)(\alpha -(\frac{-\frac{\gamma^{2}\alpha^{2}q}{AGK}-\frac{\gamma^{2}K_{p}}{AGK}-\frac{\gamma^{2}q}{EI}+\gamma^{2}\beta^{2}Z+\alpha^{2}Z}{\alpha (\gamma^{2}+Z)}+\frac{\alpha G_{P}}{AGK}+\frac{K_{p}}{\alpha (AGK)}))+\frac{\tau_{2}\sin(\alpha L)}{AGK})+A_{2}(\sin(\alpha L)(-(\frac{\frac{\gamma^{2}\alpha^{2}r}{AGK}+\frac{\gamma^{2}\;K_{p}}{AGK}+\frac{\gamma^{2}r}{EI}-\gamma^{2}\beta^{2}Z-\alpha^{2}Z}{\alpha (\gamma^{2}+Z)}-\frac{\alpha q}{AGK}-\frac{K_{p}}{\alpha (AGK)})-\alpha )+\frac{\tau_{2}\cos(\alpha L)}{AGK})+A_{3}(\cosh(\beta L)(\beta -(\frac{-\frac{\gamma^{2}\beta^{2}q}{AGK}+\frac{\gamma^{2}K_{p}}{AGK}+\frac{\gamma^{2}q}{EI}+\gamma^{2}\alpha^{2}Z+\beta^{2}Z}{q(\gamma^{2}+Z)}+\frac{\beta q}{AGK}-\frac{K_{p}}{\beta (AGK)}\;))+\frac{\tau_{2}\sinh(\beta L)}{AGK})+A_{4}((\sinh(\beta L)(\beta -(\frac{-\frac{\gamma^{2}\beta^{2}q}{AGK}+\frac{\gamma^{2}K_{p}}{AGK}+\frac{\gamma^{2}q}{EI}+\gamma^{2}p^{2}Z+q^{2}Z}{q(\gamma^{2}+Z)}+\frac{q}{AGK}-\frac{K_{p}}{q(AGK)}\;))+\frac{\tau_{2}\cosh(\beta L)}{AGK}))=0$$

(3.20) $$A_{1}(\frac{1}{EI}((\delta_{2}\cos(\alpha L)(\frac{-\frac{\gamma^{2}\alpha^{2}q}{AGK}-\frac{\gamma^{2}K_{P}}{AGK}-\frac{\gamma^{2}q}{EI}+\gamma^{2}\beta^{2}Z+\alpha^{2}Z}{\alpha (\gamma^{2}+Z)}+\frac{\alpha q}{AGK}+\frac{K_{p}}{\alpha (AGK)}\;))+(-\sin(\alpha L))(\frac{-\frac{\gamma^{2}\alpha^{2}q}{AGK}\;-\frac{\gamma^{2}K_{p}}{AGK}-\frac{\gamma^{2}q}{EI}+\gamma^{2}\beta^{2}Z+\alpha^{2}Z}{\gamma^{2}+Z}+\frac{\alpha^{2}q}{AGK}+\frac{K_{p}}{AGK}))+A_{2}(\frac{1}{EI}((\delta_{2}\sin(\alpha L))(\frac{\frac{\gamma^{2}q(\alpha^{2}-\beta^{2})}{AGK}+\frac{\gamma^{2}K_{p}}{AGK}+\frac{\gamma^{2}q}{EI}+\gamma^{2}(-\beta^{2})-\alpha^{2}Z}{\alpha (\gamma^{2}+Z)}-\frac{\alpha q}{AGK}-\frac{K_{p}}{\alpha (AGK)}))+\cos(\alpha L)(\frac{\frac{\gamma^{2}\alpha^{2}q}{AGK}+\;\frac{\gamma^{2}K_{p}}{AGK}+\frac{\gamma^{2}q}{EI}-\gamma^{2}\beta^{2}Z-\alpha^{2}Z}{\gamma^{2}+Z}-\frac{\alpha^{2}q}{AGK}-\frac{K_{p}}{AGK}))+A_{3}\frac{1}{EI}((\delta_{2}\cosh(\alpha L))(\frac{-\frac{\gamma^{2}\beta^{2}q}{AGK}+\frac{\gamma^{2}K_{p}}{AGK}+\frac{\gamma^{2}q}{EI}+\gamma^{2}\alpha^{2}+\beta^{2}Z}{\beta (\gamma^{2}+Z)}+\frac{\beta q}{AGK}-\frac{K_{p}}{\beta (AGK)}\;))+\sinh(\beta L)(\frac{-\frac{\gamma^{2}\beta^{2}q}{AGK}+\;\frac{\gamma^{2}K_{p}}{AGK}+\frac{\gamma^{2}q}{EI}+\gamma^{2}\alpha^{2}+\beta^{2}Z}{\gamma^{2}+Z}+\frac{\beta^{2}q}{AGK}-\frac{K_{p}}{AGK})+A_{4}(\frac{1}{EI}((\delta_{2}\sinh(\beta L))(\frac{-\frac{\gamma^{2}\beta^{2}q}{AGK}+\frac{\gamma^{2}K_{p}}{AGK}+\frac{\gamma^{2}q}{EI}+\gamma^{2}\alpha^{2}Z+\beta^{2}Z}{\beta (\gamma^{2}+Z)}+\frac{\beta r}{AGK}-\frac{K_{p}}{\beta (AGK)}\;))+\cosh(\beta L)(\frac{-\frac{\gamma^{2}\beta^{2}q}{AGK}+\;\frac{\gamma^{2}K_{p}}{AGK}+\frac{\gamma^{2}q}{EI}+\gamma^{2}\alpha^{2}Z+\beta^{2}Z}{\gamma^{2}+Z}+\frac{\beta^{2}q}{AGK}-\frac{K_{p}}{AGK}))=0$$

where Z and *γ* are stated as

$$\gamma =\sqrt{\frac{E}{GK}}$$

and

$$Z=\frac{q}{AGK}+1$$

The boundary conditions (2.3a)-(2.3d) yielded the findings given by Eqs. (3.17)-(3.20) show a system of four equations with four unknowns $A_{1},A_{2},A_{3}$, and $A_{4}$. As a result, a non-trivial solution requires that the coefficient matrix determinant be zero, leading to the characteristic solution. It is significant to note that the characteristic equation can be used to determine the eigenvalues *α* and *β*. The explicit values of *α* or *β* can only be found when *α* is expressed as a function of *β* or otherwise. The process outlined below is used to specifically determine the eigenvalues. The following forms are obtained by solving Eq. (3.12) for $E_{1}$, $E_{2}$, and $E_{3}$.

(3.21) $$E_{1}=\frac{\frac{Z(\alpha^{2}-\beta^{2})}{2}+\frac{q\gamma^{2}}{2EIZ}-\frac{K_{p}\gamma^{2}}{2KGAZ}}{Z+\gamma^{2}}\;E_{2}=\frac{E_{1}\gamma^{2}}{Z}+\frac{K_{p}\gamma^{2}}{2KGAZ}+\frac{q\gamma^{2}}{2EIZ^{2}}\;E_{3}=\frac{Z^{2}\alpha^{2}\beta^{2}+4\alpha^{2}\beta^{2}\gamma^{4}+4Z\gamma^{2}\alpha^{4}+4Z\gamma^{2}\beta^{4}}{4{\left(Z+\gamma^{2}\right)}^{2}}+\frac{\gamma^{2}}{Z}{\left(\frac{q\gamma^{2}}{EIZ(Z+\gamma^{2})}\right)}^{2}+\frac{2\gamma^{2}}{Z}\frac{Z(\alpha^{2}-\beta^{2})}{Z+\gamma^{2}}\frac{q\gamma^{2}}{EIZ(Z+\gamma^{2})}+\frac{\gamma^{2}}{Z{\left(Z+\gamma^{2}\right)}^{2}}{\left(\frac{K_{P}\gamma^{2}}{KGAZ}\right)}^{2}-\frac{2\gamma^{2}}{Z}\frac{2(\alpha^{2}-\beta^{2})+\frac{q\gamma^{2}}{EIZ}}{Z+\gamma^{2}}\frac{K_{P}\gamma^{2}}{KGAZ(Z+\gamma^{2})}+(\frac{\gamma^{2}K_{P}}{KGAZ^{2}}+\frac{q\gamma^{2}}{EIZ^{2}})(\frac{Z(\alpha^{2}-\beta^{2})+\frac{q\gamma^{2}}{EIZ}-\frac{K_{P}\gamma^{2}}{KGAZ}}{Z+\gamma^{2}})$$

In the following expression for *β*, we take ratio $E_{3}$ to $E_{1}$ by using Eqs. (3.12) and (3.21).

(3.22) $$\beta =\sqrt{\frac{H-M}{h\sqrt{Z}}}$$

where

$$M=\frac{1}{2\gamma \sqrt{Z}}(Z(\gamma^{2}+Z)(-\frac{qK_{p}}{A^{2}G^{2}K^{2}Z^{2}}-\frac{EK_{p}}{AG^{2}K^{2}Z^{2}}+\frac{K_{p}}{AGKZ^{2}}+\frac{s^{2}}{Z^{2}})+\frac{2\gamma^{4}K_{p}}{AGKZ}-\gamma^{2}K_{p}Z(\gamma^{2}+Z)AGKZ^{2}+\frac{2\gamma^{4}q}{EIZ}-\frac{\gamma^{2}qZ(\gamma^{2}+Z)}{EIZ}+\gamma^{4}\alpha^{2}+\alpha^{2}Z^{2})$$

$$H^{2}=((-\frac{qK_{p}}{A^{2}G^{2}K^{2}Z^{2}}-\frac{EK_{p}}{A\;G^{2}K^{2}Z^{2}}+\frac{K_{p}}{AGKZ^{2}}+\frac{s^{2}}{Z^{2}})((\gamma^{2}+Z)(-\frac{\gamma^{2}K_{p}}{AGKZ}-\frac{\gamma^{2}q}{EIZ}+\alpha^{2}Z)+\frac{K_{p}{\left(\gamma^{2}+Z\right)}^{2}}{AGKZ}+\frac{q{\left(\gamma^{2}+Z\right)}^{2}}{EIZ})-\frac{\gamma^{2}K_{p}(\gamma^{2}\;+Z)(-\frac{\gamma^{2}K_{p}}{AGKZ}-\frac{\gamma^{2}q}{EIZ}+\alpha^{2}Z)}{AGKZ^{2}}-\frac{1}{EIZ}(\gamma^{2}q(\gamma^{2}+Z)(-\frac{\gamma^{2}K_{p}}{AGKZ}-\frac{\gamma^{2}q}{\;}EIZ+\alpha^{2}Z))-\frac{{\left(\gamma^{2}+Z\right)}^{2}(qK_{p})}{AEIGKZ^{2}}+\frac{2\gamma^{4}K_{p}(\alpha^{2}Z-\frac{\gamma^{2}q}{EIZ})}{AGKZ^{2}}-\frac{Z}{\gamma^{2}{\left(\frac{\gamma^{2}K_{p}}{AGK\gamma }\right)}^{2}}+\frac{2\gamma^{2}p^{2}\;(\gamma^{2}q)}{EIZ}-\frac{\gamma^{2}{\left(\frac{\gamma^{2}q}{EIZ}\right)}^{2}}{Z}-\frac{K_{p}{\left(\gamma^{2}+Z\right)}^{2}}{EIZ^{2}}-\gamma^{2}\alpha^{4}Z)+M^{2}$$

and

$$s=L\sqrt{\frac{A}{I}}$$

where *s* is known as slenderness ratios. The characteristic equation for the Timoshenko beam depends on both *γ* and *s*. Thus, the eigenvalues depend on both physical and geometrical properties. According to the above procedure the characteristic equation, along with Eq. (3.22), yields *α* and *β* using the root finding procedure. The eigenfrequencies can also be determined using Eq. (3.21). It is essential to note that characteristic equations for shear and Rayleigh models are derived by ignoring the effects of shear deformation and rotary inertia, respectively, from Eqs. (2.1a)-(2.1b). As a special case, the characteristic equation for Euler-Bernoulli beams can also be derived by removing both rotary and shear deformations from coupled equations (2.1a)-(2.1b).

### 3.2 Finite element formulation

The GFM starts by dividing the Timoshenko beam into finite elements. The weight functions $w_{1}$ and $w_{2}$ both have corresponding values for *ν* and *ψ*, which are multiplied by the differential equation (2.1a)-(2.1b) to obtain weak forms.

(3.23) $$\int_{0}^{L}-w_{1}\left(\rho A\nu_{tt}-KGA(\nu_{xx}-\psi_{x})+K_{p}\nu -q\nu_{xx}\right)dx=\int_{0}^{L}w_{1}\rho A\nu_{tt}dx-w_{1}KGA\left(\nu_{x}-\psi \right){|}_{0}^{L}+\int_{0}^{L}w_{1,x}KGA(\nu_{x}-\psi )dx+\int_{0}^{L}K_{p}w_{1}\nu dx-w_{1}q\nu_{x}{|}_{0}^{L}+\int_{0}^{L}w_{1,x}q\nu_{x}dx=0$$

(3.24) $$\int_{0}^{L}-w_{2}\left(EI\psi_{xx}+KGA(\nu_{x}-\psi )+\rho I\psi_{tt}\right)dx=-w_{2}EI\left(\psi_{x}\right){|}_{0}^{L}+\int_{0}^{L}w_{2,x}EI\psi_{x}dx+\int_{0}^{L}w_{2}KGA\left(\psi -\nu_{x}\right)dx-\int_{0}^{L}\rho Iw_{2}\left(\psi_{tt}\right)dx=0$$

The interpolation functions for *ν* and *ψ* need to be introduced after obtaining the weak form for coupled differential equations. Assume that *ν* and *ψ* have general quadratic and cubic interpolation shape functions given in Eqs (3.25)–(3.26), respectively:

(3.25) $$\nu =\left[1\;\;\;\left(\frac{x}{L}\right)\;\;\;{\left(\frac{x}{L}\right)}^{2}\;\;\;{\left(\frac{x}{L}\right)}^{3}\right]\left[\begin{array}{cccc} 1\; & 0\; & 0\; & 0\\ b_{11}\; & b_{21}\; & b_{31}\; & b_{41}\\ b_{12}\; & b_{22}\; & b_{32}\; & b_{42}\\ b_{13}\; & b_{23}\; & b_{33}\; & b_{43} \end{array}\right]\left[\begin{array}{c} \nu_{1}\\ \psi_{1}\\ \nu_{2}\\ \psi_{2} \end{array}\right]$$

and

(3.26) $$\psi =\left[1\;\;\;\left(\frac{x}{L}\right)\;\;\;{\left(\frac{x}{L}\right)}^{2}\right]\left[\begin{array}{cccc} 0\; & 1\; & 0\; & 0\\ c_{11}\; & c_{21}\; & c_{31}\; & c_{41}\\ c_{12}\; & c_{22}\; & c_{32}\; & c_{42} \end{array}\right]\left[\begin{array}{c} \nu_{1}\\ \psi_{1}\\ \nu_{2}\\ \psi_{2} \end{array}\right]$$

**Figure 4:**
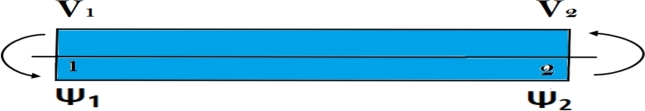
Timoshenko beam element.

The coefficients $b_{ij}$ and $c_{ij}$ are undefined, and $\nu_{1}$, $\psi_{1}$, $\nu_{2}$, and $\psi_{2}$ are the nodal displacements and rotations at the beam ends 1 and 2, respectively (see Fig. 4). By requiring that $\left(\nu (x=L)=\nu_{2}\right)$ and $(\psi (x=L)=\psi_{2}$), four of the $b_{ij}$ and four of the $c_{ij}$ coefficients can be calculated in term of the remaining twelve (12) coefficients. Substituting the shape functions into the Eqs. (2.2a)-(2.2b) and solving them yield the remaining coefficients. The two shape functions are presented in Eqs. (3.27) and (3.28), respectively:

(3.27) $${\left[N_{\nu }\right]}^{T}=\left[\begin{array}{c} \frac{1}{1+\left\{\phi \right\}}\left\{2\;\frac{x^{3}}{L^{3}}-3\;\frac{x^{2}}{L^{2}}-\frac{\left\{\phi \right\}x}{L}+1+\left\{\phi \right\}\right\}\\ \frac{L}{1+\left\{\phi \right\}}\left\{\frac{x^{3}}{L^{3}}-\frac{x^{2}}{L^{2}}\left(2+\frac{\left\{\phi \right\}}{2}\right)+\frac{x}{L}\left(1+\frac{\left\{\phi \right\}}{2}\right)\right\}\\ -\frac{1}{1+\left\{\phi \right\}}\left\{2\;\frac{x^{3}}{L^{3}}-3\;\frac{x^{2}}{L^{2}}-\frac{\left\{\phi \right\}x}{L}\right\}\\ \frac{L}{1+\left\{\phi \right\}}\left\{\frac{x^{3}}{L^{3}}-\frac{x^{2}}{L^{2}}\left(1-\frac{\left\{\phi \right\}}{2}\right)-\frac{\left\{\phi \right\}x}{2\;L}\right\} \end{array}\right]$$

(3.28) $${\left[N_{\psi }\right]}^{T}=\left[\begin{array}{c} \;\frac{6}{\left(1+\left\{\phi \right\}\right)L}\left\{\frac{x^{2}}{L^{2}}-\frac{x}{L}\right\}\\ \frac{1}{1+\left\{\phi \right\}}\left\{\;\frac{x^{2}}{L^{2}}-\frac{\left(4+\left\{\phi \right\}\right)x}{L}+1+\left\{\phi \right\}\right\}\\ -\;\frac{6}{\left(1+\left\{\phi \right\}\right)L}\left\{\frac{x^{2}}{L^{2}}-\frac{x}{L}\right\}\\ \frac{1}{1+\left\{\phi \right\}}\left\{\;\frac{3x^{2}}{L^{2}}-\frac{\left(2-\left\{\phi \right\}\right)x}{L}\right\} \end{array}\right]$$

here *ϕ* is defined as:

$$\phi =\frac{12EI}{L^{2}KGA}$$

**Figure 5:**
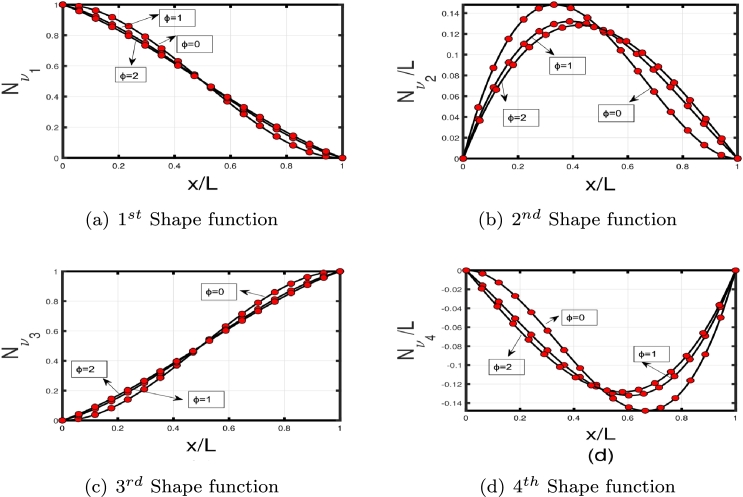
Shape function variation with *ϕ* for transverse displacements.

**Figure 6:**
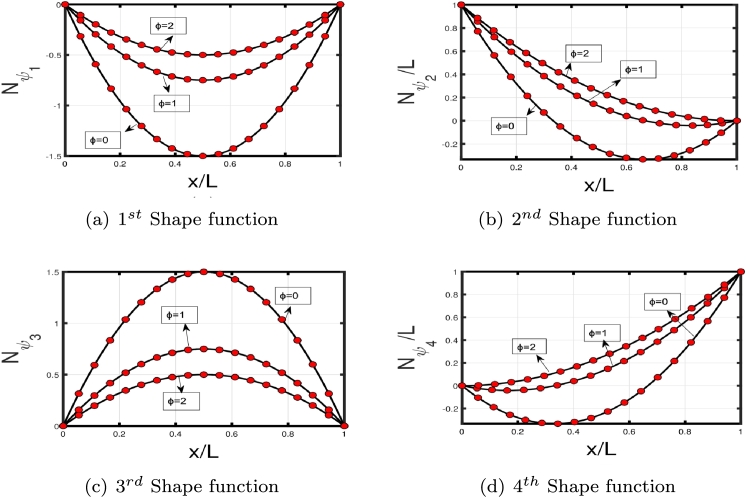
Shape functions variation with *ϕ* for rotational displacement.

It is intriguing to consider that the shape functions rely on *ϕ*, as illustrated in Figure 5, Figure 6 (a)–(d). In the case of long slender beams (ϕ=0), $\left[N_{\nu }\right]$ reduces to the cubic Hermitian polynomial and $\left[N_{\psi }\right]$ reduces to the derivative of $\left[N_{\nu }\right]$. Although for composite or short beams, the polynomials are exclusively computed by a particular value of *ϕ*. By substituting Eqs. (3.27)–(3.28) into Eqs. (3.23)–(3.24), we get

(3.29) $$\int_{0}^{L}-w_{1}\left(\rho A\nu_{tt}-KGA(\nu_{xx}-\psi_{x})+K_{p}-q\nu_{xx}\right)dx=\int_{0}^{L}\lambda_{i}\rho A\lambda_{j}\nu_{j,tt}dx-\lambda_{i}KGA(\nu_{j}\lambda_{j,x}-\psi_{m}\lambda_{m}){|}_{0}^{L}+\int_{0}^{L}\lambda_{i,x}KGA\left(\nu_{j}\lambda_{j,x}-\psi_{m}\lambda_{m}\right)dx+\int_{0}^{L}K_{p}\lambda_{i}\nu_{j}\lambda_{j}dx-\lambda_{i}q\nu_{j}\lambda_{j,x}{|}_{0}^{L}+\int_{0}^{L}\lambda_{j,x}q\left(\nu_{j}\lambda_{j,x}\right)dx=0$$

(3.30) $$\int_{0}^{L}-w_{2}\left(EI\psi_{xx}+KGA(\nu_{x}-\psi )+\rho I\psi_{tt}\right)dx=-\lambda_{k}EI\lambda_{m,x}\left(\psi_{m}\right){|}_{0}^{L}+\int_{0}^{L}\lambda_{k,x}EI\psi_{m}\lambda_{m,x}dx+\int_{0}^{L}\lambda_{k}KGA\left(\psi_{m}\lambda_{m}-\nu_{j}\lambda_{j,x}\right)dx-\int_{0}^{L}\rho I\lambda_{k}\left(\psi_{m,tt}\right)dx=0$$

Equations (3.29)-(3.30) can be expressed as

(3.31) $$[k_{ij}]\nu_{j}+[m_{ij}]\nu_{j,tt}=0$$

where

$$k_{ij}=\int_{0}^{L}\lambda_{i,x}KGA(\lambda_{j,x}\nu_{j}-\psi_{m}\lambda_{m})dx+\int_{0}^{L}K_{f}\lambda_{j}\lambda_{i}\nu_{j}dx+\int_{0}^{L}\lambda_{j,x}G_{P}\lambda_{j,x}\nu_{j}dx+\int_{0}^{L}\lambda_{k,x}EI\psi_{m}\lambda_{m,x}dx+\int_{0}^{L}\lambda_{k}KGA(\psi_{m}\lambda_{m}-\nu_{j}\lambda_{j,x})dx$$

and

$$m_{ij}=\int_{0}^{L}\nu_{j,tt}\lambda_{i}\rho A\lambda_{j}dx-\int_{0}^{L}\rho I\lambda_{k}\left(\psi_{m,tt}\right)dx$$

where $k_{ij}$ and $m_{ij}$ are the stiffness and mass matrices, respectively. Therefore, we write harmonic time dependent $\nu_{j}$ as follows:

(3.32) $$\nu_{j}=\left\{\overset{¯}{\nu_{j}}\right\}e^{i\omega t}$$

Substituting Eq. (3.32) into Eq. (3.31), we obtain

(3.33) $$[k_{ij}]-\omega^{2}[m_{ij}]=0$$

MATLAB code is developed on the basis of FEM to calculate the eigenfrequencies and eigenmodes of the beam subject to elastic constraints. The global stiffness matrices can be constructed straightforwardly with MATLAB for the highest number of elements. From the stiffness matrix, foundation stiffness matrix, and mass matrix, eigenvalues can be obtained using Eq. (3.33), where eigenfrequencies are calculated through the square root of eigenvalues [30], [32], [33]. Moreover, the derived stiffness element can be reduced to the stiffness matrix associated with an Euler-Bernoulli beam for long beams with ϕ=0, which suggests it is free of shear locking. As $m_{ij}$ and $k_{ij}$ dependent on *ϕ*, all of these matrices can be simplified to the standard Euler-Bernoulli based form by letting ϕ=0.

## 4 Results and discussions

This section is focused on demonstrating the modal analysis of elastically constrained beams including the Timoshenko beam, shear beam, Rayleigh beam, and Euler Bernoulli beam. These beams are supported by different types of foundations, such as Winkler, Pasternak, and Hetényi. The beams being examined have uniform cross-sectional dimensions. The primary objective of this section is to showcase the modal behavior of these beams and their response to external forces when subjected to various types of foundations. The uniform beam's dimensions and physical characteristics have been taken from reference [31]. The following parameters are provided: L=1 m, A=0.0097389 m^2^, $E=200\times 10^{9}$ Pa, I=0.0001171 m^4^, ρ=7830 kg/m^3^, K=0.53066, and G=77.5 GPa, respectively.

**Figure 7:**
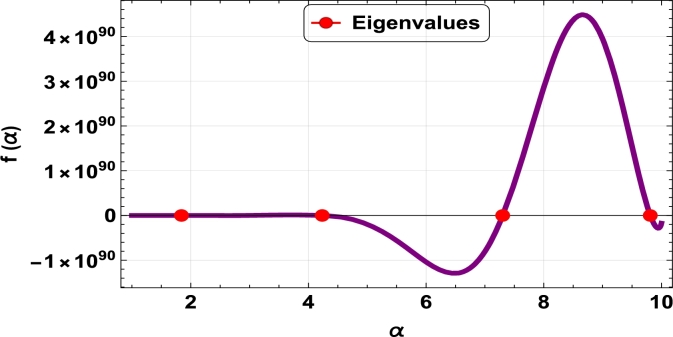
Eigenvalues for TB resting over Pasternak foundation for *τ*_1_ = 10^12^,*δ*_1_ = 10^13^,*τ*_2_ = *δ*_2_ = 0 and *K*_*p*_ = *G*_*P*_ = 10^5^.

**Figure 8:**
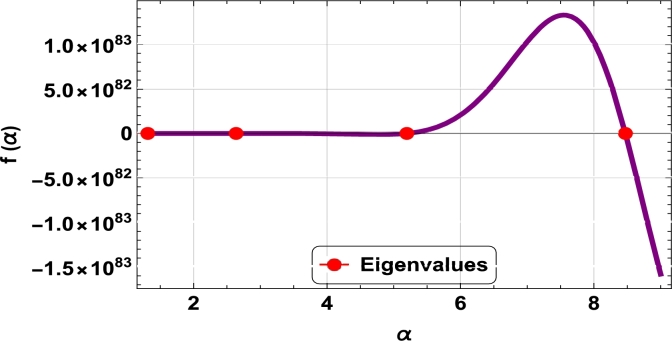
Eigenvalues for TB resting over Winkler foundation for *τ*_1_ = *δ*_2_ = 10^8^,*τ*_2_ = *δ*_1_ = 10^2^ and *K*_*p*_ = 10^7^.

**Figure 9:**
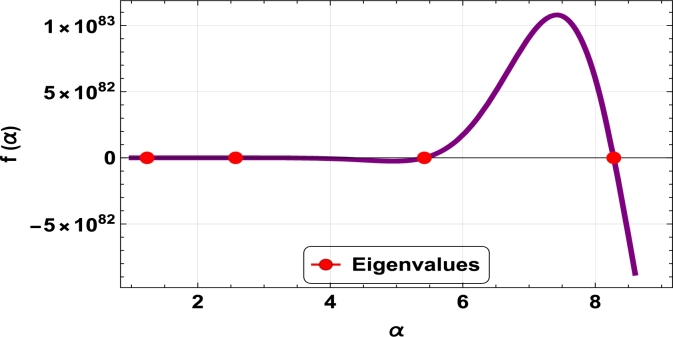
Eigenvalues for TB resting over Hetényi foundation for *δ*_1_ = *δ*_2_ = 10^8^,*τ*_1_ = *τ*_2_ = 10^2^ and *K*_*p*_ = 10^10^.

**Figure 10:**
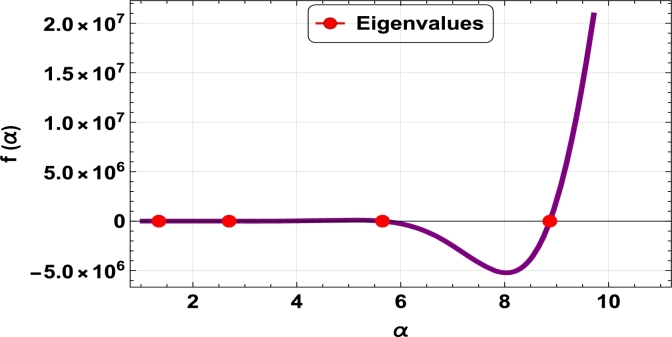
Eigenvalues of SB resting over Winkler foundation for *δ*_1_ = *δ*_2_ = 10^8^,*τ*_1_ = *τ*_2_ = 10^2^, *K*_*p*_ = 10^6^.

**Figure 11:**
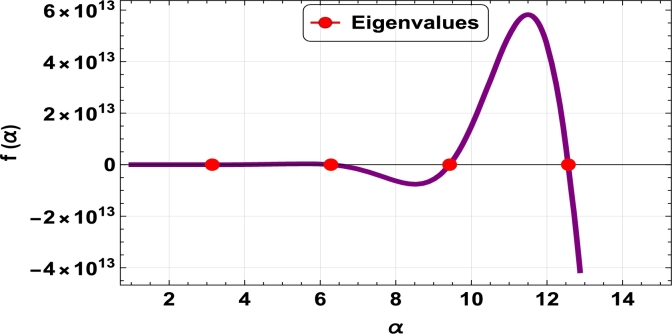
Eigenvalues for SB resting over Hetényi foundation for *δ*_1_ = *δ*_2_ = 10^11^,*τ*_1_ = *τ*_2_ = 0, *K*_*p*_ = 10^5^.

**Figure 12:**
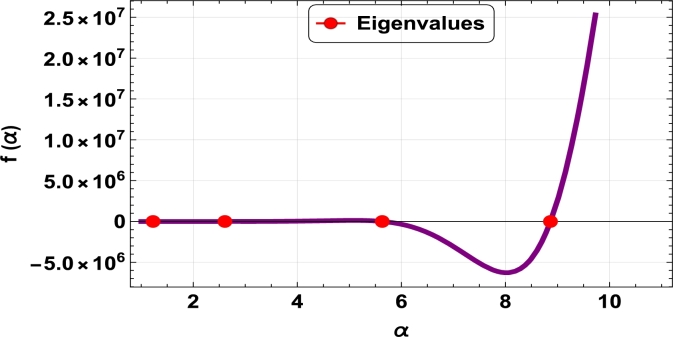
Eigenvalues for SB resting over Pasternak foundation for *δ*_1_ = *δ*_2_ = 10^8^,*τ*_1_ = *τ*_2_ = 10^2^ and *Kp* = *G*_*P*_ = 10^6^.

**Figure 13:**
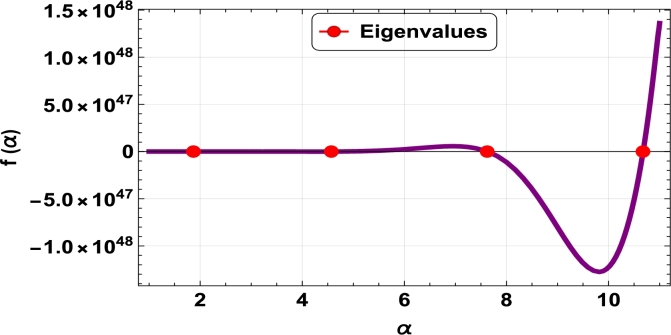
Eigenvalues of beam for RB resting over Pasternak foundation for *δ*_1_ = 10^13^,*τ*_1_ = 10^12^,  *δ*_2_ = *τ*_2_ = 0 and *G*_*P*_ = *K*_*p*_ = 10^5^.

**Figure 14:**
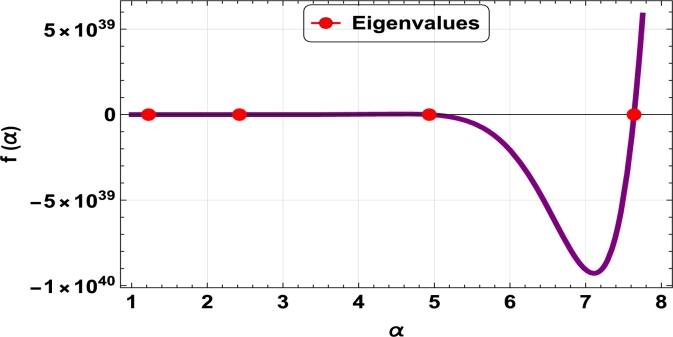
Eigenvalues of beam for RB resting over Hetényi foundation for *τ*_1_ = *δ*_2_ = 10^8^,*τ*_2_ = *δ*_1_ = 10^2^ and *K*_*p*_ = 10^5^.

**Figure 15:**
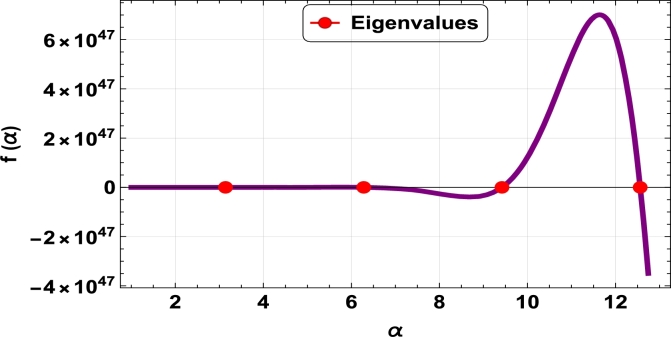
Eigenvalues of beam for RB resting over Winkler foundation for *δ*_1_ = *δ*_2_ = 10^11^,*τ*_1_ = *τ*_2_ = 0 and *K*_*p*_ = 10^7^.

The dispersive relations of elastically constrained TB, SB, and RB over single-parameter elastic foundations (Winkler) and two-parameter elastic foundations (Pasternak and Hetényi) are represented by zeros (eigenvalues) in Figure 7, Figure 8, Figure 9, Figure 10, Figure 11, Figure 12, Figure 13, Figure 14, Figure 15. By calculating the eigenvalues, the corresponding eigenfrequencies can be determined to identify the mode. Thus, the figures illustrate the eigenvalues and their respective eigenfrequencies for the different types of beams and foundations.

Table 1 provides a comparison of the first four initial eigenfrequencies of TB, SB, RB, and EBB supported by the Winkler foundation ($K_{p}=10^{5}=10^{6}=10^{7}$) with the parameters $\delta_{1}=\delta_{2}=10^{11},\tau_{1}=\tau_{2}=0$. When eigenfrequencies are compared to analytical results, the GFM is shown to accurately anticipate them, demonstrating its appropriateness for real-world applications. A Winkler foundation is happened to increase eigenfrequencies because of the extra stiffness it gives the beam, which is an important realization for structural design. Notably, shear and Timoshenko beam models' natural frequencies closely reflect those of the Euler-Bernoulli and Rayleigh models, highlighting how crucial it is to choose the right beam model in order to comprehend and anticipate eigenfrequencies.

**Table 1:** The eigenfrequencies of EBB, RB, SB, and TB resting over the Winkler foundation.

BC	*K*_*p*_	$\delta_{1}=\delta_{2}=10^{11},\tau_{1}=\tau_{2}=0$			
		*ω*_1_	*ω*_2_	*ω*_3_	*ω*_4_
TB-AM	10^5^	676.7842	1869.9960	3098.3151	4307.7885
FEM		676.6466	1870.0907	3099.1446	4278.7078
PE		0.02%	0.005%	0.02%	0.6%

SB-AM		693.2353	1914.4500	3148.4500	4354.3207
FEM		693.0408	1914.4940	3149.0744	4356.7945
PE		0.02%	0.002%	0.01%	0.05%

RB-FEM		822.6827	2866.0579	5445.4897	8176.9109

EBB-FEM		870.1290	3480.4454	7830.9953	13921.77089

TB-AM	10^6^	678.7842	1870.9699	3099.0125	4308.3257
FEM		676.8569	1870.1666	3099.1911	4278.7095
PE		0.2%	0.04%	0.03%	0.6%

SB-AM		695.5743	1917.6072	3152.4974	4359.7722
FEM		693.2561	1914.5723	3149.1216	4356.8288
PE		0.3%	0.1%	0.1%	0.06%

RB-FEM		822.845	2866.0933	5445.5030	8176.9172

EBB-FEM		870.3008	3480..4884	7831.0143	13921.7816

TB-AM	10^7^	683.8948	1880.9342	3106.2049	4313.8170
FEM		678.9561	1870.9254	3099.6558	4278.7262
PE		0.7%	0.5%	0.2%	0.8%

SB-AM		700.5461	1948.8986	3194.8004	4413.9167
FEM		695.4064	1935.3530	3169.5960	4457.1719
PE		0.7%	0.6%	0.7%	0.9%

RB-FEM		824.4674	2866.4469	5445.6357	8176.9802

EBB-FEM		872.01668	3480.9178	7831.2052	13921.8889

**Figure 16:**
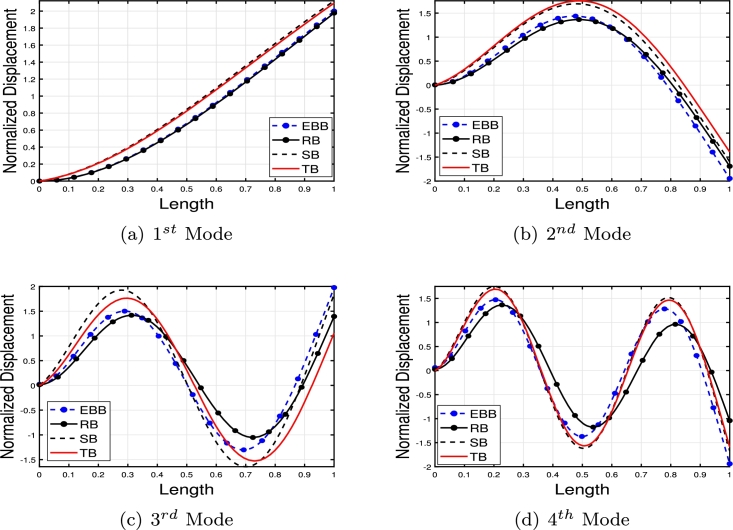
The comparison of lowest four eigenmodes of beams for *τ*_1_ = 10^12^,*δ*_1_ = 10^13^,  *τ*_2_ = *δ*_2_ = 0.

**Figure 17:**
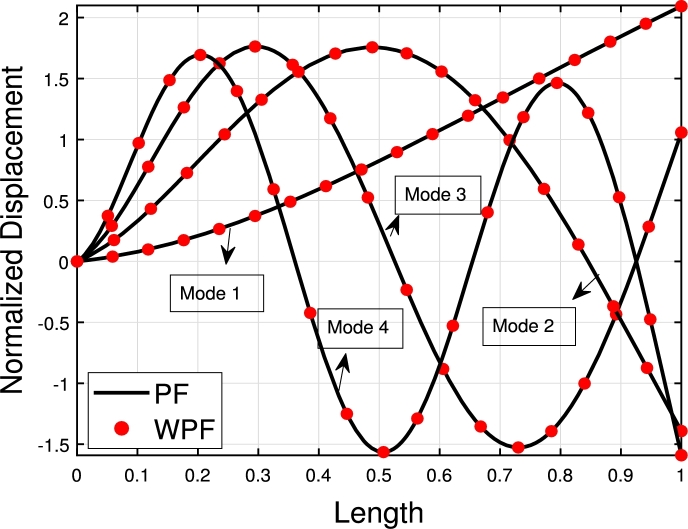
The lowest four eigenmodes of TB resting over Pasternak foundation by letting *τ*_1_ = 10^12^,  *δ*_1_ = 10^13^,*δ*_1_ = *τ*_2_ = 0,*K*_*p*_ = *G*_*P*_ = 10^5^.

**Figure 18:**
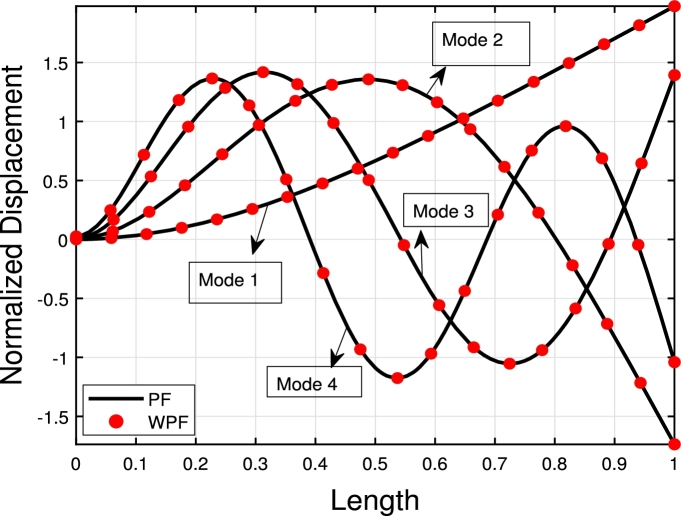
The lowest four eigenmodes of RB resting over Pasternak by letting *τ*_1_ = 10^12^, *δ*_1_ = 10^13^,*δ*_2_ = *τ*_2_ = 0, *G*_*P*_ = *K*_*p*_ = 10^7^.

Table 2 presents a comparison of the eigenfrequencies of TB, SB, RB, and EBB supported by the Pasternak foundation ($G_{P}=10^{5}=10^{6}=10^{7}$) with $\tau_{1}=10^{12},\delta_{1}=10^{13},\tau_{2}=\delta_{2}=0$. The results show that FEM produces the most accurate agreement with the analytical solution. The eigenfrequencies increase with an increase in the shear foundation modulus $\left(G_{P}\right)$. A comparison of these beams reveals that the presence of shear deformation in the shear model, rotary inertia effects in the Rayleigh model, and both shear deformation and rotary inertia in Timoshenko models result in a significant reduction in eigenfrequencies. For EBB, the addition of rotating inertia effects, shear deformation, and both shear deformation and rotating inertia effects reduces the eigenfrequencies by up to 2%, 15%, 27%, 62%, 11%, 40%, 55%, 65%, and 12%, 44%, 58%, 69%, respectively. Therefore, incorporating both shear deformation and rotary inertia has a greater impact on the eigenfrequencies of EBB compared to incorporating only one of these effects. It is also worth noting that the clamped-free eigenfrequencies for TB, SB, RB, and EBB agree well with those of Han et al. [31] when $\tau_{1}=10^{12},\delta_{1}=10^{13},\tau_{=}\delta_{1}=0$. Fig. 16(a)–(d) shows a comparison of the initial four modes of TB, SB, RB, and EBB with $\tau_{1}=10^{12},\delta_{1}=10^{13},\tau_{1}=\delta_{1}=0$. Notably, EBB and RB show analogous eigenmodes, implying analogous vibration patterns across both models. Similar to EBB and RB, SB and TB also share same eigenmodes. Furthermore, in Fig. 17, TB is supported by a Pasternak foundation, its eigenmodes are unaffected by shear and rotating inertia but their associated eigenfrequencies are increased, indicating that the foundation is crucial in modifying the stiffness and natural frequencies of the beam. By contrasting the eigenmodes of RB with and without a Pasternak foundation, Fig. 18 further emphasizes the impact of the foundation. These results offer useful insights into the behavior of beams under various circumstances and open up new directions for future studies on structural dynamics and design optimization.

**Table 2:** The eigenfrequencies of EBB, RB, SB, and TB resting over the Pasternak foundation.

BC	*G*_*P*_	*K*_*p*_	$\delta_{1}=10^{13},\tau_{1}=10^{12},\;\delta_{2}=\tau_{1}=0$			
	*ω*_1_	*ω*_2_	*ω*_3_	*ω*_4_		
TB-AM	0	0	269.9090	1076.8437	2269.8871	3248.5407
TB-FEM	0	0	269.9093	1076.8753	2270.2058	3249.2542
PE			0.001%	0.002%	0.01%	0.02%

SB-AM			274.5145	1150.4065	2409.8510	3633.4331
FEM			275.1687	1150.5749	2410.5269	3635.2941
PE			0.2%	0.01%	0.02%	0.05%

RB-AM			301.7452	1646.1920	3932.1504	6527.2319

EBB-FEM			310.1204	1941.3951	5425.9845	10602.0590

TB-AM	10^5^	10^5^	269.9743	1076.6781	2270.0708	3248.4375
FEM			270.2426	1077.2600	2270.6197	3249.6370
PE			0.09%	0.05%	0.02%	0.03%

SB-AM			274.7811	1150.6942	2410.2493	3633.9593
FEM			275.3264	1150.9382	2411.0078	3635.9269
PE			0.2%	0.02%	0.03%	0.05%

RB-FEM			302.1264	1646.5171	3932.3711	6527.4039

EBB-FEM			310.5023	1941.7760	5426.3038	10602.3570

TB-AM	10^6^	10^6^	272.0469	1079.9594	2270.9360	3249.1105
FEM			273.2167	1080.7154	2274.3406	3253.0752
PE			0.4%	0.004%	0.07%	0.1%

SB-AM			277.1688	1153.2807	2413.8309	3638.6908
FEM			278.1700	1154.6800	2415.0362	3640.9706
PE			0.3%	0.1%	0.04%	0.06%

RB-FEM			305.5317	1649.4410	3934.3569	6528.9514

EBB-FEM			313.9722	1945.2064	5429.1774	1060.0389

TB-AM	10^7^	10^7^	299.1757	1110.9819	2299.7490	3255.9794
FEM			302.7823	1114.6615	2311.1961	3286.7614
PE			0.5%	0.3%	0.4%	0.9%

SB-AM			300.0031	1178.8328	2449.3590	3685.6717
FEM			305.5699	1190.4816	2455.3107	3691.8341
PE			0.5%	0.9%	0.2%	0.1%

RB-FEM			337.35353	1678.4138	3954.1735	6544.4098

EBB-FEM			346.4227	1979.1454	5457.8300	10631.8205

Table 3 compares the eigenmodes of different beams (TB, SB, RB, and EBB) supported by a two-parametric foundation, namely the Hetényi foundation. The analysis is conducted by setting $\tau_{1}=\tau_{2}=0$, $\delta_{1}=\delta_{2}=10^{11}$, and $K_{p}=0,10^{5},10^{10}$. The results show that when $K_{p}=10^{5}$, the eigenfrequencies of TB, SB, RB, and EBB decrease, whereas the eigenfrequencies increase when $K_{p}=10^{10}$. This implies that the effect of the Hetényi elastic foundation on the natural frequency of the beam depends on the relative stiffness of the beam and foundation. When the foundation is much stiffer than the beam, the natural frequency of the beam increases, indicating that the beam becomes more rigid and less prone to vibration. Conversely, if the foundation is much softer than the beam, the natural frequency of the beam decreases, indicating that the beam becomes more flexible and more prone to vibration.

**Table 3:** The eigenfrequencies of EBB, RB, SB, and TB resting over Hetényi the foundation.

BC	*K*_*p*_	$\delta_{1}=\delta_{2}=10^{11},\tau_{1}=\tau_{2}=0$			
		*ω*_1_	*ω*_2_	*ω*_3_	*ω*_4_
TB-AM	0	676.6147	1869.8980	3097.8467	4307.0027
FEM		676.6232	1870.0823	3099.1394	4278.7067
PE		0.001%	0.01%	0.04%	0.6%

SB-AM		693.1561	1914.0988	3147.7620	4353.7146
FEM		693.01558	1914.4854	3149.0687	4353.7146
PE		0.02%	0.02%	0.04%	0.07%

RB-FEM		822.6646	2866.0540	5445.4883	8176.9102

EBB-FEM		870.1100	3480.4406	7830.9931	13921.7696

TB-AM	10^5^	646.6193	1848.0899	3082.4034	4241.1800
FEM		643.9362	1840.9640	3070.4062	4199.7407
PE		0.4%	0.1%	0.3%	0.9%

SB-AM		629.0172	1830.4126	3036.2284	4212.4381
FEM		635.20625	1832.1407	3036.8320	4210.2804
PE		0.9%	0.09%	0.01%	0.05%

RB-AM		764.2465	2816.5672	5403.9364	8141.9152

EBB-FEM		808.3227	3420.3456	7771.2387	13862.1882

TB-AM	10^10^	1852.6773	2544.7213	3553.1212	4599.0148
FEM		1861.7915	2527.1321	3519.3736	4582.6547
PE		0.4%	0.6%	0.9%	0.3%

SB-AM		1930.0819	2584.273	3541.0760	4587.8307
FEM		1928.0521	2583.0447	3541.2414	4589.8084
AE		0.1%	0.03%	0.004%	0.04%

RB-AM		1891.3376	3191.4807	555.5627	8211.9852

EBB-FEM		1993.7691	3875.6281	7982.0975	13981.4874

**Figure 19:**
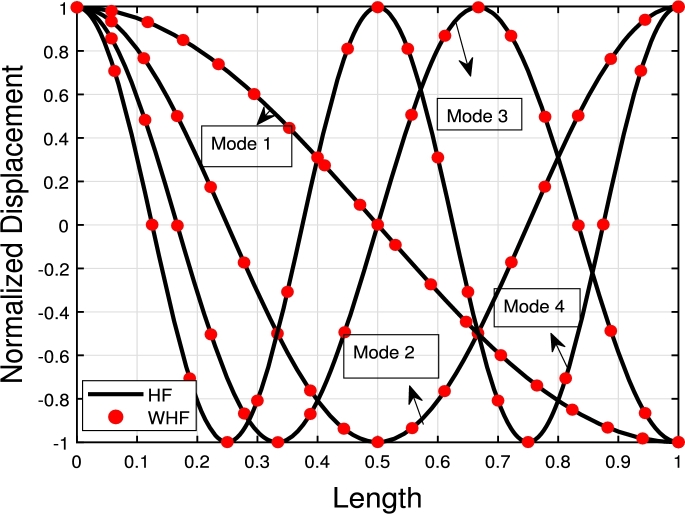
The lowest four eigenmodes of TB resting over Hetényi foundation by letting *τ*_1_ = *τ*_2_ = 0,*δ*_1_ = *δ*_2_ = 10^11^,*K*_*p*_ = 10^5^.

**Figure 20:**
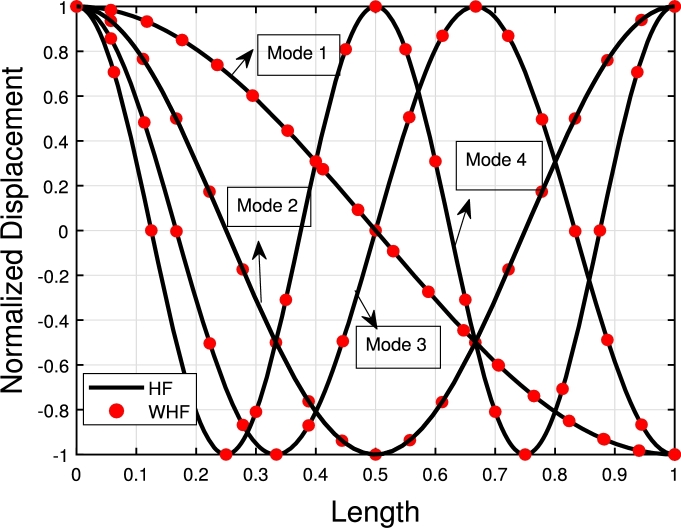
The lowest four eigenmodes of SB resting over Hetényi foundation by letting *τ*_1_ = *τ*_2_ = 0,*δ*_1_ = *δ*_2_ = 10^11^,*K*_*p*_ = 10^10^.

**Figure 21:**
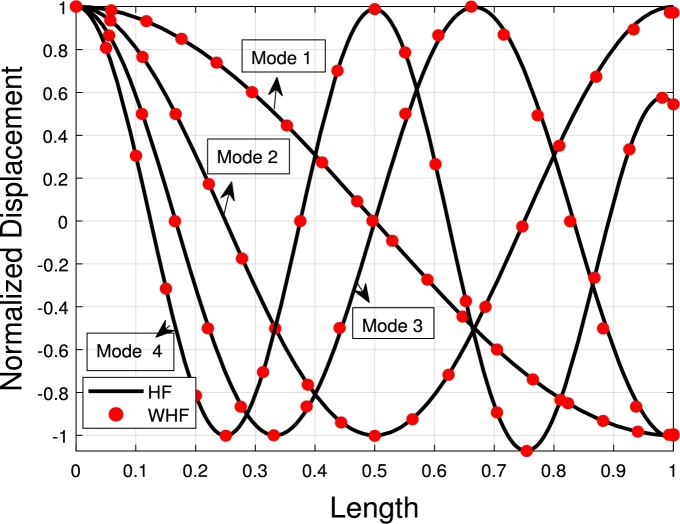
The lowest four eigenmodes of RB resting over Hetényi foundation by letting *τ*_1_ = *τ*_2_ = 0,*δ*_1_ = *δ*_2_ = 10^11^,*K*_*p*_ = 10^10^.

Figure 19, Figure 20, Figure 21 illustrate the comparison of the lowest four eigenmodes of TB, SB, and RB with and without Hetényi Foundation (WHF). It is important to note that the flexural rigidity factor does not affect the eigenmodes, but it does reduce or enhance the corresponding eigenfrequencies accordingly.

**Figure 22:**
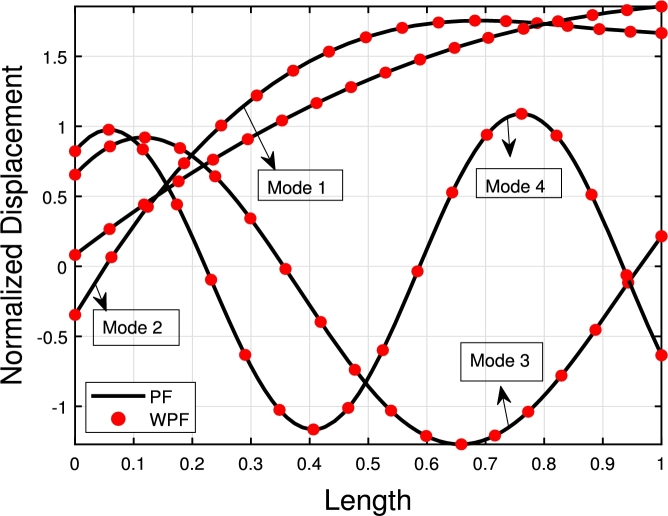
The lowest four eigenmodes of SB resting over Pasternak foundation by letting *τ*_1_ = *δ*_2_ = 10^8^,*δ*_1_ = *τ*_2_ = 10^2^,*G*_*P*_ = *K*_*p*_ = 10^5^.

The values presented in Table 4, Table 5, Table 6 show the eigenfrequencies of TB, SB, RB, and EBB placed over Winkler, Pasternak, and Hetényi foundations, respectively. The parameters used in these tables are $\delta_{2}=\tau_{1}=10^{8}$ and $\delta_{1}=\tau_{2}=10^{2}$, which result in similar behavior for natural frequencies and solution accuracy as seen in Table 1, Table 2, Table 3. The tables demonstrate that the presence of Winkler and Pasternak foundations increases the eigenfrequencies, as the beam becomes stiffer in the presence of a shear layer and elastic stiffness. Additionally, the eigenfrequencies of the beams over the Pasternak foundation are higher than those over the Winkler foundation. Fig. 22 illustrates the SB eigenmodes with and without the Pasternak foundation.

**Table 4:** The eigenfrequencies of EBB, RB, SB, and TB resting over the Winkler foundation.

BC	*K*_*p*_	$\delta_{2}=\tau_{1}=10^{8},\;\delta_{1}=\tau_{2}=10^{2}$			
		*ω*_1_	*ω*_2_	*ω*_3_	*ω*_4_
TB-AM	10^5^	127.9510	485.0074	1532.0074	2652.6667
FEM		127.9510	485.1251	1532.6072	2652.6667
PE		0%	0.2%	0.001%	0.004%

SB-AM		128.4361	508.9177	1656.6247	2931.0318
FEM		128.4361	508.9172	1656.6247	2930.5529
PE		0%	0.00009%	0%	0.01%

RB-FEM		131.0915	510.6261	1888.3151	3946.4149

EBB-FEM		131.6364	532.6364	2361.7548	5918.5107

TB-AM	10^6^	129.1052	485.4040	1532.6658	2652.5972
FEM		129.1051	485.4046	1532.6888	2652.7079
PE		0.00007%	0.0001%	0.001%	0.004%

SB-AM		129.5938	509.2037	1656.5761	2930.6039
FEM		129.5938	509.2101	1656.7147	2931.0827
PE		0%	0.06%	0.008%	0.01%

RB-FEM		132.2173	1510.8401	1888.3657	3946.4318

EBB-FEM		132.7671	533.2486	2361.8181	5918.5360

TB-AM	10^7^	140.1248	488.1902	1533.4815	2653.0093
FEM		140.1248	488.1908	1533.5045	2653.1199
PE		0.0001%	0.0001%	0.001%	0.004%

SB-AM		140.6561	512.1309	1657.4782	2931.1139
FEM		140.6557	512.1352	1657.6196	2931.9093
PE		0.0002%	0.0008%	0.008%	0.02%

RB-FEM		142.9897	522.6605	1888.8710	3946.6014

EBB-FEM		143.5858	536.0443	2362.4509	5918.7887

**Table 5:** The eigenfrequencies of EBB, RB, SB, and TB resting over the Pasternak foundation.

BC	*G*_*P*_	*K*_*p*_	$\delta_{2}=\tau_{1}=10^{8},\;\delta_{1}=\tau_{2}=10^{2}$			
			*ω*_1_	*ω*_2_	*ω*_3_	*ω*_4_
TB-AM	10^5^	10^5^	128.1051	485.2012	1533.1086	2652.3683
FEM			128.0697	485.5038	1533.0949	2653.4393
PE			0.02%	0.1%	0.008%	0.04%

SB-AM			128.5578	509.0226	1656.7788	2930.9913
FEM			128.5535	509.3170	1657.1767	2932.1673
PE			0.003%	0.05%	0.2%	0.03%

RB-FEM			131.2102	502.4226	1888.6727	3946.6763

EBB-FEM			131.7545	533.4226	2362.1985	5918.9035

TB-AM	10^6^	10^6^	130.3468	485.8379	1537.5345	2656.3013
FEM			130.2595	485.1650	1536.9388	2657.4438
PE			0.06%	0.1%	0.03%	0.04%

SB-AM			131.4518	511.3146	1659.7298	2935.0479
FEM			130.7362	513.1704	1661.2688	2936.9537
PE			0.5%	0.3%	0.09%	0.06%

RB-FEM			133.3705	506.0950	1891.9381	3949.0442

EBB-FEM			133.9059	537.7767	2366.2510	5922.4612

TB-AM	10^7^	10^7^	153.8165	492.3759	1581.6970	2695.1780
FEM			154.6018	493.6018	1582.8145	2696.9714
PE			0.1%	0.2%	0.07%	0.06%

SB-AM			151.3885	523.2001	1686.5021	2974.6283
FEM			152.5282	525.3715	1687.5729	2975.5229
AE			0.7%	0.4%	0.06%	0.03%

RB-FEM			151.9379	545.0969	1924.2874	3972.6502

EBB-FEM			152.3876	579.7684	2406.3723	5957.9114

**Table 6:** Eigenfrequencies of EBB, RB, SB, and TB resting over the Hetényi foundation.

BC	*K*_*p*_	$\delta_{2}=\tau_{1}=10^{8},\;\delta_{1}=\tau_{2}=10^{2}$			
		*ω*_1_	*ω*_2_	*ω*_3_	*ω*_4_
TB-AM	0	127.8578	485.1307	1532.6168	2652.5986
FEM		127.82221	485.0940	1532.5982	2652.6621
PE		0.02%	0.007%	0.001%	0.002%

SB-AM		128.3067	508.8775	1656.4759	2930.5473
FEM		128.3067	508.8844	1656.6146	2931.0261
PE		0%	0.001%	0.008%	0.01%

RB-FEM		130.9658	510.9658	1888.3095	2931.0261

EBB-FEM		131.5126	532.9372	2361.7478	5918.5080

TB-AM	10^5^	69.7245	454.8466	1508.6080	2634.9375
FEM		69.4594	454.1151	1518.9228	2646.4644
PE		0.3%	0.1%	0.02%	0.4%

SB-AM		70.4636	455.7625	1580.2033	2824.3044
FEM		70.1550	455.8637	1588.3496	2804.9205
PE		0.4%	0.02%	0.5%	0.06%

RB-FEM		75.7396	457.0310	1849.1232	3912.4655

EBB-FEM		76.6892	486.8767	2255.2531	5825.7699

TB-AM	10^10^	1766.5160	1840.7693	2185.2977	2993.2516
FEM		1763.4883	1833.8198	2179.5261	2977.5457
PE		0.1%	0.3%	0.2%	0.5%

SB-AM		1825.0306	1885.1834	2416.2853	3362.7421
FEM		1823.8669	1867.9241	2425.4695	3345.0398
PE		0.06%	0.06%	0.3%	0.5%

RB-FEM		1838.9405	1878.2884	2594.5988	4314.2374

EBB-FEM		1843.9841	1899.8857	2899.6372	6104.2055

**Figure 23:**
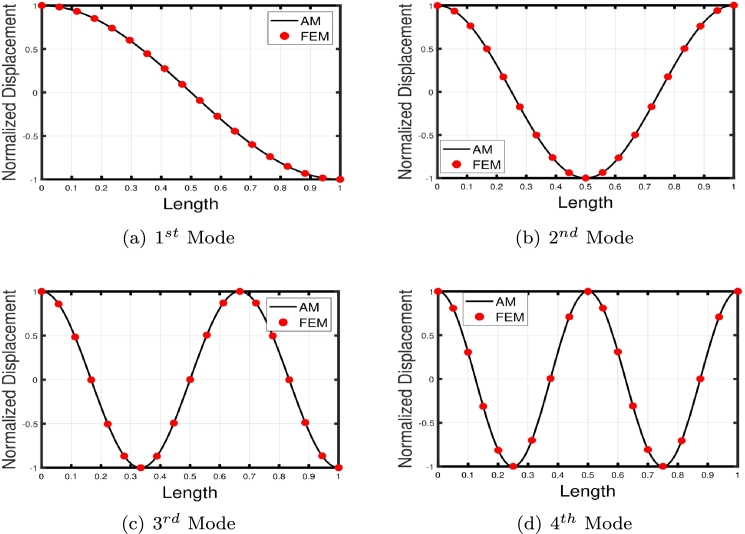
The comparison of lowest four eigenmodes of SB resting over Hetényi foundation by letting *τ*_1_ = *τ*_2_ = 0,*δ*_1_ = *δ*_2_ = 10^11^,*K*_*p*_ = 10^10^.

**Figure 24:**
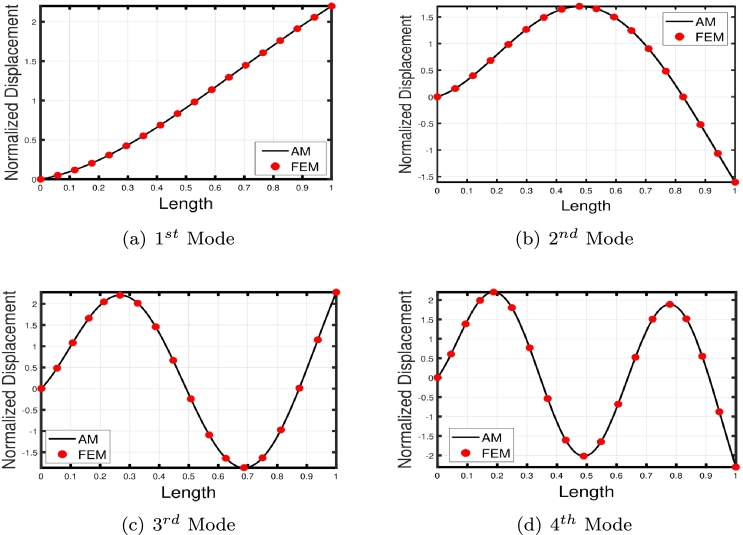
The comparison of lowest four eigenmodes of SB resting over Pasternak *τ*_1_ = 10^12^, *δ*_1_ = 10^13^,*δ*_2_ = *τ*_2_ = 0, *G*_*P*_ = *K*_*p*_ = 10^7^.

**Figure 25:**
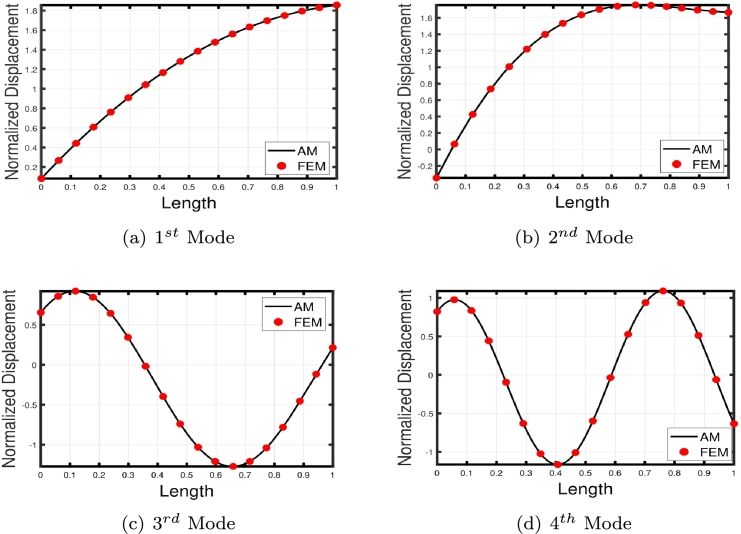
The comparison of lowest four eigenmodes of SB resting over Pasternak foundation by letting *τ*_1_ = *δ*_2_ = 10^8^,*δ*_1_ = *τ*_2_ = 10^2^,*K*_*p*_ = 10^5^.

**Figure 26:**
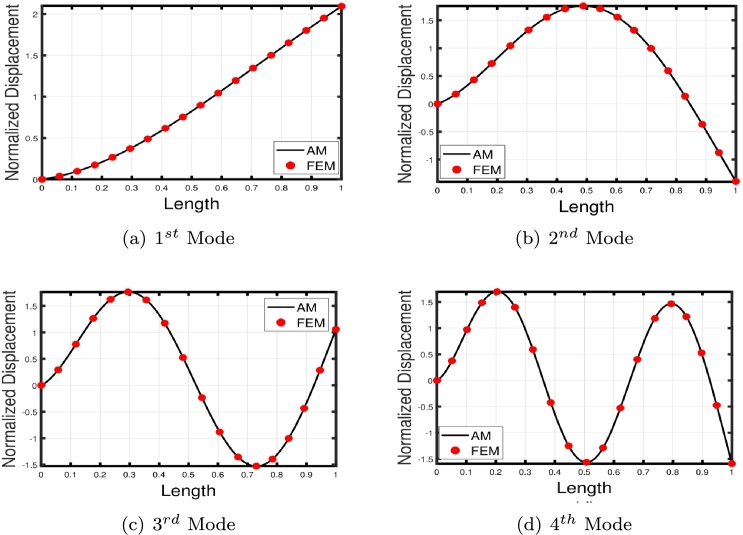
The comparison of lowest four eigenmodes of TB over Pasternak *τ*_1_ = 10^12^, *δ*_1_ = 10^13^,*δ*_2_ = *τ*_2_ = 0, *G*_*P*_ = *K*_*p*_ = 10^5^.

The comparison between the numerical and analytical results for SB and TB supported by the Hetényi and Pasternak foundations is illustrated in Figure 23, Figure 24, Figure 25, Figure 26 (a)–(d). These graphs demonstrate that the numerical and analytical results exhibit a high degree of similarity.

Based on the above analysis, it is clear that the natural frequency of a beam can be controlled by adjusting the elastic foundation parameter. This can help to minimize collateral damage to the vibrating structure. The results show that the presence of a Winkler or Pasternak foundation can enhance the eigenfrequencies of the beam, making it stiffer and less prone to vibration. However, the magnitude of this effect depends on the relative stiffness of the beam and the foundation. The analytical and numerical results also show great agreement, confirming the validity of the analytical models. Overall, these findings highlight the importance of considering elastic foundations in the design of vibrating structures to ensure their safety and longevity. Thus, placing the beam over an elastic foundation can help control its vibration, which may otherwise cause damage to the overall structure.

## 5 Conclusions

The objective of this research was to investigate the natural frequencies of finite beams with different types of foundations using various beam models, namely Timoshenko, shear, Rayleigh, and Euler-Bernoulli beam models. We analyzed the effect of physical properties and geometry on the characteristic equation and eigenfrequencies of the different models. The findings revealed that the Timoshenko and shear models have similar eigenfrequencies, while the Rayleigh and Euler-Bernoulli models also have similar eigenfrequencies. However, the shear deformation and rotary inertia in the Timoshenko model had a greater influence on the eigenfrequencies compared to rotary inertia in the Rayleigh model and shear deformation in the shear model. Additionally, the natural frequencies of the beam increased due to the shear layer, flexural rigidity, and foundation constant. We observed that the Hetényi elastic foundation influenced the natural frequency of the beam based on the relative magnitudes of the beam stiffness and foundation stiffness. It was also found that the results showed excellent agreement with the finite element results for the first four eigenmodes and eigenfrequencies. This suggests that the finite element scheme is an excellent technique for obtaining accurate eigenmodes and mode shapes for similar problems.

Based on these research findings, we recommend considering more complex problems related to higher beam theories and modified Timoshenko models. Further research should also focus on investigating the effects of other types of foundations and boundary conditions on the eigenfrequencies and mode shapes of beams. We believe that this study can contribute to the development of better techniques for predicting the dynamic behavior of structures, which is crucial for ensuring their safety and reliability.

## CRediT authorship contribution statement

**Gulnaz Kanwal:** Writing – original draft, Software, Resources, Methodology, Investigation, Formal analysis, Conceptualization. **Naveed Ahmed:** Writing – review & editing, Validation, Supervision, Software, Project administration, Funding acquisition, Data curation. **Rab Nawaz:** Writing – review & editing, Validation, Supervision, Project administration, Methodology, Investigation, Formal analysis, Conceptualization.

## Declaration of Competing Interest

The authors declare that they have no known competing financial interests or personal relationships that could have appeared to influence the work reported in this paper.

## Data Availability

No additional data is required because all the data essential to produce the findings is already included in the article.
